# Electrochemical Sensor for Tryptophan Determination Based on Trimetallic-CuZnCo-Nanoparticle-Modified Electrodes

**DOI:** 10.3390/molecules29010028

**Published:** 2023-12-20

**Authors:** Adina Arvinte, Ana-Lacramioara Lungoci, Adina Coroaba, Mariana Pinteala

**Affiliations:** “Petru Poni” Institute of Macromolecular Chemistry, Centre of Advanced Research in Bionanoconjugates and Biopolymers, Grigore Ghica Voda Alley 41A, 700487 Iasi, Romaniaadina.coroaba@icmpp.ro (A.C.); pinteala@icmpp.ro (M.P.)

**Keywords:** trimetallic nanoparticles, CuZnCo, electrodeposition, tryptophan detection

## Abstract

The superior properties of electrodeposited trimetallic CuZnCo nanoparticles, arising from the synergistic effect of combining the unique features of metallic components, were confirmed using voltametric measurements. The surface morphology and structure of the as-prepared electrocatalysts were determined using scanning electron microscopy, energy-dispersive X-ray, and X-ray photoelectron spectroscopy techniques. Here, the trimetallic CuZnCo nanoparticles were synthesized as a powerful redox probe and highly efficient signal amplifier for the electrochemical oxidation of tryptophan. Differential pulse voltammetry studies showed a linear relationship with a tryptophan concentration of 5–230 μM, and the low detection limit was identified at 1.1 μM with a sensitivity of 0.1831 μA μM^−1^ cm^−2^.

## 1. Introduction

Tryptophan (Trp) is an α-amino acid that is essential in the biosynthesis of proteins in humans. As a naturally occurring indole, Trp plays distinct roles as a biochemical precursor for the neurotransmitter serotonin, the hormone melatonin, and vitamin B3 [1]. Acute tryptophan deficiency is associated with increased pain sensitivity, anxiety and irritability, the acoustic startle response, motor activity, and aggression in humans, while high levels of L-Trp can cause side effects such as drowsiness, stomach pain, vomiting, diarrhea, headache, and blurry vision [2]. Because of its biological importance, Trp is indispensable in human nutrition since it cannot be biologically synthesized. The Trp content of food is therefore a very important factor for the growth and general health and function of animals as well as humans [3]. 

Various chromatographic approaches to the determination of tryptophan and its metabolites in biological tissues and fluids include high-performance liquid chromatography (HPLC) with several detection modalities, such as mass spectrometry, UV absorbance, and fluorescence and electrochemistry [4,5,6,7,8,9,10]. A major challenge in Trp analysis is that the acidic conditions used to hydrolyze protein for normal amino acid analysis causes substantial or even complete oxidative degradation of Trp, which means that Trp can hardly be quantified [11]. Therefore, alkaline hydrolysis is typically used to partially solve this problem. Recently, electrochemical detection has found many applications in the determination of electroactive amino acids due to the inherent electroactivity of their thiol or aromatic groups. Moreover, because of the double-bond of indolyl, Trp can be easily oxidized to form a C–N double bond via the electrochemical route. Thus, electrochemical methods are commonly used in the detection of tryptophan, since its oxidation behavior has been studied for decades [12,13]. Brabec and Mornstein [12] showed that Trp oxidized electrochemically on a graphite electrode, yielding an unstable intermediate that reacts and thus produces unidentified products.

Malfoy and Reynaud [13] also reported that Trp oxidized electrochemically on gold and carbon electrodes in two well-developed anodic peaks. It was suggested that a two-electron oxidation process of tryptophan at the graphite electrode occurred at the double bond in the pyrrole ring rather than the benzene ring, particularly at low concentrations (0.2 mM of amino acid), given the fact that the benzene ring of the indoles is less reactive than the pyrrole ring substituted at position 3. At higher bulk concentrations (2 mM), the peak current is limited due to the adsorption of amino acids.

Chemical modification of electrodes with suitable materials was a prior consideration devised to address the electrochemical oxidation of Trp [14]. Different metal-nanostructure-based electrodes like Ag@C core–shell nanoparticles [15], flower-like cerium vanadate [16], cerium oxide nanoparticles on reduced graphene oxide [17], copper–cobalt hexacyanoferrate [18], Nafion/TiO_2_-graphene [19], polydopamine/graphene/MnO_2_ composite [20], MnWO_4_-nanoplate-encapsulated graphene oxide nanocomposite [21], Cu_2_O-reduced graphene oxide nanocomposite [22], and Pd–Cu@Cu_2_O particles [23] have been developed for the sensing of Trp.

Over the past few years, multimetallic nanoparticles (Nps) have drawn more attention because of their distinct properties compared to monometallic nanoparticles, thus increasing their applications in various fields. Bimetallic NPs have already become a very popular topic for electrochemical detection because of their strong ability to increase electronic conductivity, effective electrochemical activity, and various morphologies [18,21,23,24,25,26,27,28,29]. To further enhance conductivity and electroactivity, hybrid nanoparticles can be coupled with nanocarbon structures, like carbon nanotubes [30,31,32,33,34], graphene [35,36,37,38], and carbon black or carbon fiber [39,40,41]. Likewise, trimetallic Nps have attracted much attention owing to their unique synergistic electronic effect, useful for chemical reactions in sensors and enhancing catalytic performance [42,43,44,45].

At present, Cu-based nanomaterials are the most used catalysts for tryptophan electro-oxydation, proving to have high electrocatalytic activity, good chemical stability, and the ability to be easily prepared and functionalized [18,22,23,45]. Also, Co-based materials have attracted intensive research interest concerning their role as promising catalysts for Trp [18,46,47]. It has been proved that hydroxy groups formed on Co can efficiently capture H atoms from tryptophan molecule [46]. Extensive research has been conducted on Zn-based catalysts to improve the electrocatalytic performance of sensors. ZnO is an n-type semiconductor material with a wide bandgap (3.37 eV) that is highly popular and widely applied as an electrocatalyst. Hybrid materials have been developed by combining ZnO with p-type semiconductors, such as copper oxide [48], Ni, [49] or Co [50,51], mainly to improve the electronic and chemical effects of the surface, improving their application as sensors.

Consequently, tri-metallic Cu-based nanoparticles, for instance, Cu-Zn-Co, aroused our attention. The goal of this work was to employ electrochemical techniques in the electrosynthesis of CuZnCo trimetallic nanoparticles anchored directly to a carbon electrode and to systematically investigate the electrooxidation of tryptophan.

## 2. Results

### 2.1. Electrochemical, Morphological, and Structural Characterization of Deposited Metallic Nanoparticles

**Figure 1:**
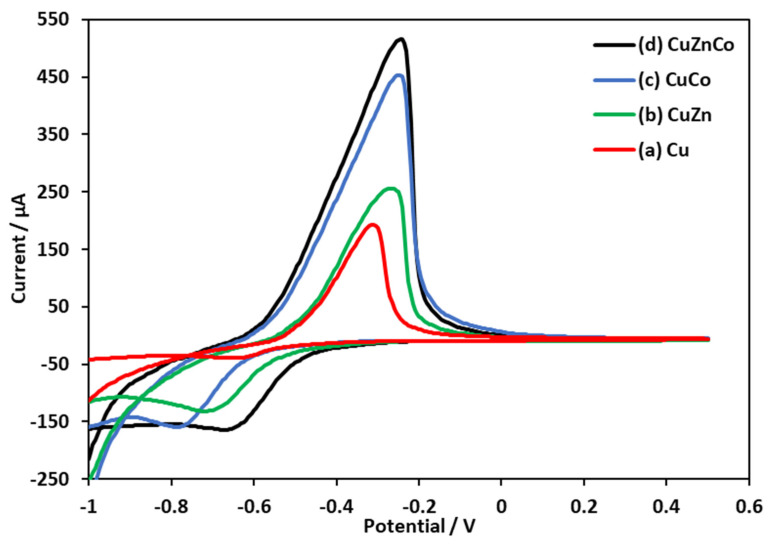
Cyclic voltammograms of (a) CuNp-, (b) CuZnNp-, (c) CuCoNp-, and (d) CuZnCoNp-modified electrodes in 0.1 M H_2_SO_4_ solution; scan rate: 0.1 V/s.

The electron transfer ability of various multicomponent-modified electrodes was evaluated according to the redox peak current of the CV curves. The electrochemical activity of the as-prepared CuNp-, CuZnNp-, CuCoNp-, and CuZnCoNp-modified electrodes was investigated by cycling the potential between −1 V and 0.5 V in a 0.1 M H_2_SO_4_ aqueous solution (Figure 1). The peak current of CuZnNp (curve b; i_ox_ = 256 μA) and CuCoNp (curve c; i_ox_ = 453 μA) increased prominently compared to that of CuNp (curve a; i_ox_ = 193 μA), showing accelerated electron transfer when copper was co-deposited with the two other metals, Zn and Co, respectively. For CuZnCoNp, the current signals increased further (curve d; i_ox_ = 516 μA), demonstrating that the trimetallic nanoparticles possess excellent conductivity due to the synergistic effect of the involved components.

**Figure 2:**
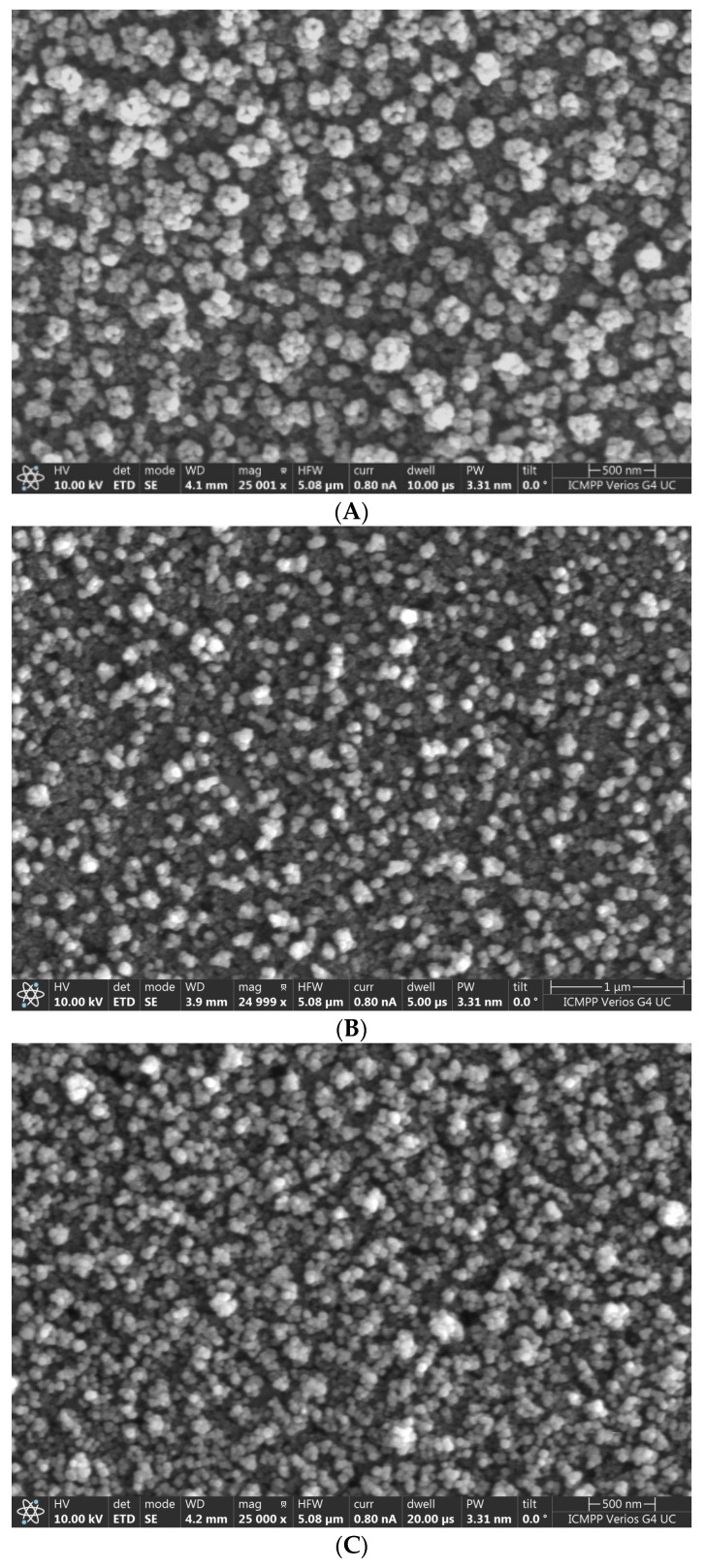

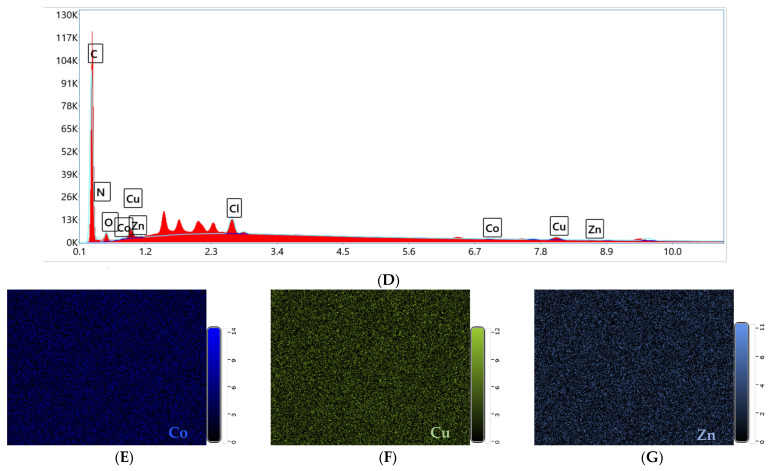
SEM images of electrodeposited nanoparticles: (**A**) CuZnCo, (**B**) CuCo, and (**C**) CuZn. Deposition conditions of nanoparticles: −1.2 V, 60 s. (**D**) EDX spectrum together with the corresponding element-mapping images of (**E**) Co, (**F**) Cu, and (**G**) Zn.

The study of the surface morphology and distribution of the nanoparticles was performed using scanning electron microscopy (SEM) along with simultaneous elemental analysis via energy-dispersive X-ray spectroscopy (EDX). Figure 2A–C show the SEM images obtained for the as-prepared CuZnCo trimetallic nanoparticles compared to bimetallic CuCo and CuZn. In the case of trimetallic electrodeposition, very dense nanoparticles cover the electrode surface as a layer with a porous structure. For bimetallic electrodeposition, the formed nanoparticles are either less dense (CuCo) or smaller (CuZn). The EDX spectrum for trimetallic nanoparticles (Figure 2D) confirms the presence of higher loadings of Cu along with Zn and Co (besides C from the electrode ink, and Cl and O remained from precursors solution). Meanwhile, the element-mapping images in Figure 2E–G confirm the spatial arrangement of Co, Cu, and Zn, distributed uniformly on the surface of the carbon electrode.

**Figure 3:**
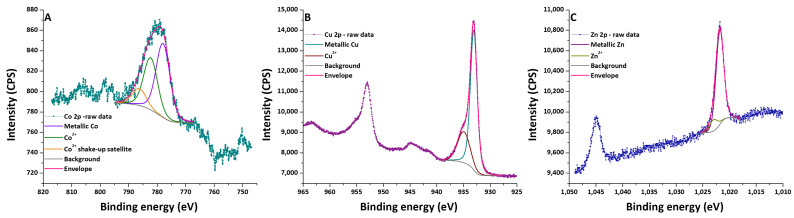
XPS high-resolution spectra of Co 2p (**A**), Cu 2p (**B**), and Zn 2p (**C**).

X-ray photoelectron spectroscopy (XPS) is a very effective method for studying the surface chemistry of metallic nanoparticles, as well as their oxidation state. The XPS analysis provided evidence about the composition of the trimetallic CuZnCo nanoparticles by revealing the presence of copper, zinc, and cobalt (Figure 3). The high-resolution spectrum of Co 2p (Figure 3A) was analyzed and resolved into three signals: metallic Co at 777.9 eV, Co^2+^ at 782.3 eV, and a Co^2+^ shake-up satellite at 786.3 eV [52]. The Cu 2p signal is shown in Figure 3B, displaying two distinct peaks at 933.1 eV and 934.9 eV. These peaks correspond to metallic copper and Cu^2+^, respectively, as indicated by their particular energy values [53]. In addition to cobalt and copper, the XPS analysis revealed the presence of zinc, as seen in Figure 3C. The Zn 2p spectra were analyzed and deconvoluted into two distinct components with specific positions at 1021.8 eV and 1022.9 eV. The initial signal may be ascribed to metallic Zn, whilst the small contribution from the high binding energy can be assigned to the Zn^2+^ oxidation state [54].

**Figure 4:**
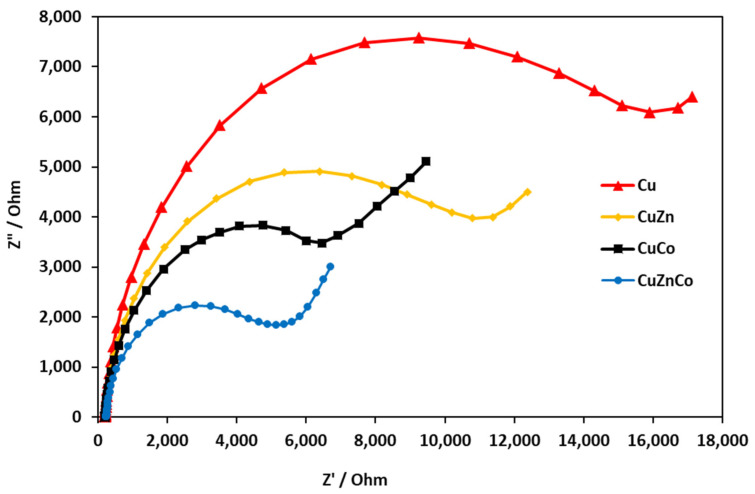
EIS measurements for CuNp-, CuZnNp-, CuCoNp-, and CuZnCoNp-modified electrodes in 5.0 mM K_3_Fe(CN)_6_/K_4_Fe(CN)_6_ (1:1) containing 0.1 M KCl. The frequency was in the range of 1–200,000 Hz.

EIS was used to evaluate the interfacial properties of the modified electrodes, and the typical Nyquist diagrams of mono-, bi-, and trimetallic-based electrodes are shown in Figure 4. Basically, the Nyquist plots consist of a semicircular part at higher frequencies and a linear part at lower frequencies characteristic of the electron transfer process. The linear part is associated with electrochemical behavior limited by diffusion, and the semicircle part reflects the electrochemical process related to electron transfer. The diameter of the semicircle corresponds to the electron transfer resistance on the electrode interface.

It is shown in Figure 4 that the charge transfer resistance of the bimetallic nanoparticles CuZn and CuCo decreased when compared to monometallic CuNp. Further, for the CuZnCo-modified electrode, the resistance is about three times less than that of the CuNp-modified electrode, suggesting that the trimetallic material shows good electricity-conducting properties. The EIS results are consistent with those obtained using CV, and both confirm accelerated electron transfer when the three metals are combined.

### 2.2. Electrocatalytic Measurement of Tryptophan 

**Scheme 1:**
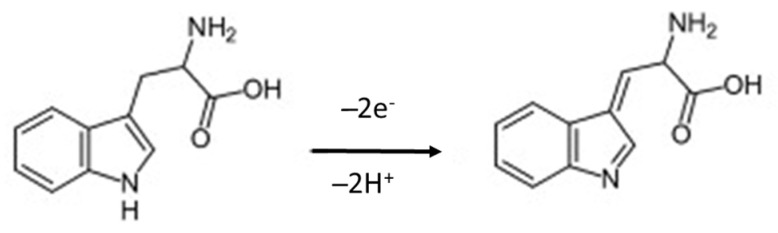
The suggested mechanism for electrochemical oxidation of L-tryptophan.

**Figure 5:**
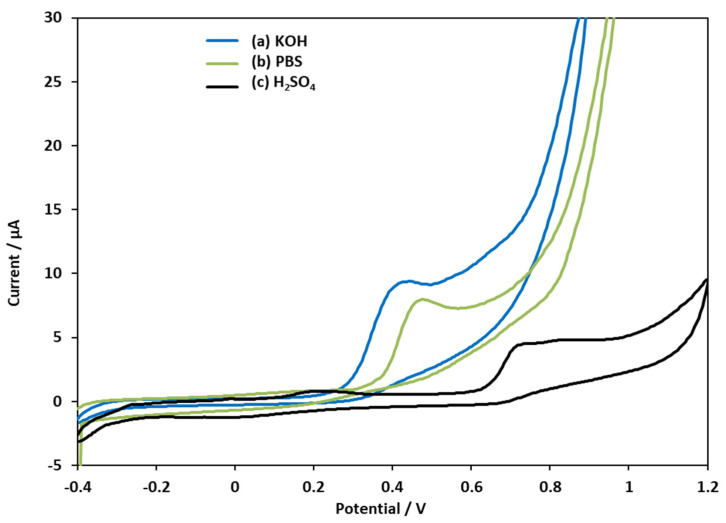
Electrochemical oxidation of 0.2 mM Trp on bare electrode in (a) basic, (b) neutral, and (c) acidic media. Scan rate: 0.1 V/s.

Tryptophan contains an α-amino group, an α-carboxylic acid group, and an indole side chain, the last of which is responsible for its specific reactivity and susceptibility to oxidative cleavage [55]. Even though the reaction mechanism of amino acids on an electrode surface is still a controversial issue, it is usually assumed that the electrochemical oxidation of Trp is an irreversible 2 e^−^ process [56,57,58,59,60], as suggested in Scheme 1, and its oxidation in aqueous solutions was investigated for a wide pH range using either glassy carbon, graphite, or boron-doped diamond electrodes. In order to evaluate the electrochemical behavior of Trp on a screen-printed carbon electrode, cyclic voltammetry measurements were made in media with different pHs. Figure 5 shows curves obtained for 0.2 mM Trp in 0.1 M H_2_SO_4_ (pH 1), 0.1 M PBS solution (pH 7), and 0.05 M KOH (pH 12) on a carbon surface electrode.

A well-defined oxidation peak can be observed at a potential of +0.5 V in PBS that shifts to lower potentials with the increase in pH (+0.46 V in KOH) and to higher potentials for smaller pH values (+0.75 V in H_2_SO_4_). Such behavior is in agreement with the results presented in the literature [56,57,59] concerning the variations in Ep with pH for the oxidation of Trp. Furthermore, from our experiment, it is obvious that the height of the oxidation peak exhibits a similar variation, with the best intensity of the current occurring in basic media. Consequently, we used the basic-pH electrolyte for the further evaluation of Trp oxidation using trimetallic nanoparticles.

**Figure 6:**
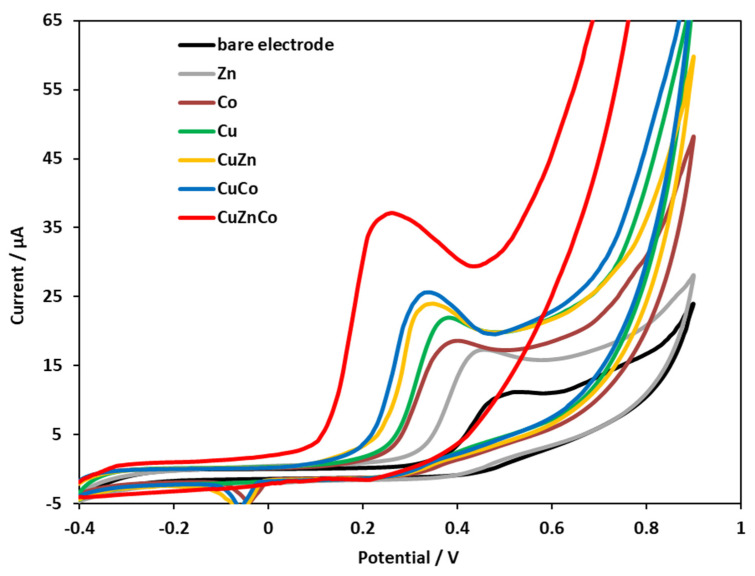
Electrochemical oxidation of 0.25 mM Trp in 0.05 M KOH (pH 12) on trimetallic-, bimetallic-, and monometallic-nanoparticle-modified electrode compared to bare electrode. Scan rate: 0.1 V/s.

Cyclic voltammograms were recorded to examine the electrocatalytic properties of the trimetallic-CuZnCo-nanoparticle-modified electrode towards 0.25 mM Trp in 0.05 M KOH, using bimetallic CuZn and CuCo nanoparticles as reference materials (Figure 6). Also, the response of monometallic nanoparticles to the oxidation of Trp was evaluated and presented comparatively.

All electrodes showed improved voltametric responses compared to the bare electrode, which can be ascribed to the mediated electrochemical oxidation of Trp on the metal-based electrode surface. The efficiency and advantages of trimetallic nanoparticles compared to bimetallic and monometallic nanoparticles with respect to the electrocatalytic oxidation of Trp are summarized in Table 1.

Thus, the shift in potential to less-negative values along with the increase in peak current demonstrates the improved electrocatalytic activity of multimetallic structures toward Trp oxidation due to their intrinsic synergistic effect that further promotes electronic transfer.

**Figure 7:**
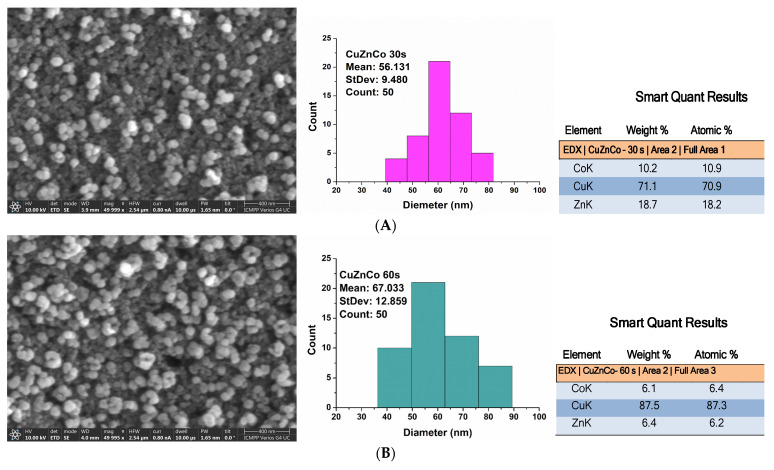

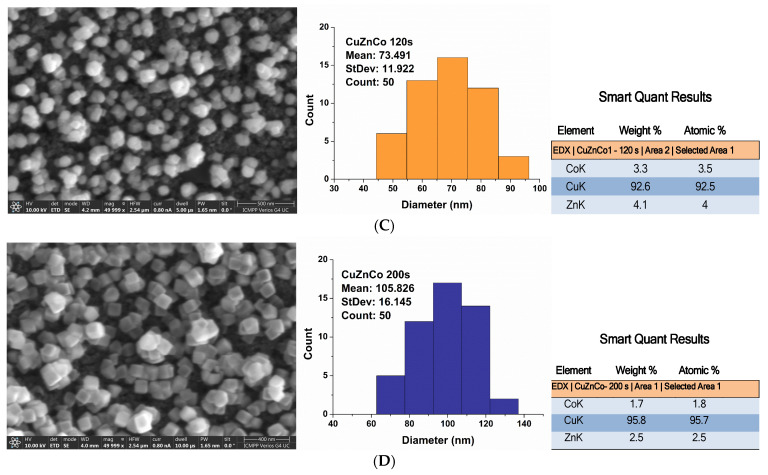
SEM images of CuZnCo nanoparticles obtained after different deposition times, namely, (**A**) 30 s; (**B**) 60 s; (**C**) 120 s; and (**D**) 200 s, accompanied by their particle size distributions and chemical compositions analyzed using energy-dispersive spectroscopy.

To optimize the method and conditions for obtaining a good electrocatalytic response to Trp, the influence of the deposition time of CuZnCo nanoparticles was evaluated. Four depositions at −1.2 V of applied potential and the same concentrations (as in Table 4) but variable times of deposition (30 s, 60 s, 120 s, and 200 s) were conducted. Figure 7 presents SEM images of the obtained nanometer-sized CuZnCo nanoparticles along with their particle size distribution and chemical composition. The morphologies of the products are spherical at short deposition times and become cubic as the deposition time reaches 200 s. The particle size distribution is very even, and the mean diameters of the spherical CuZnCo nanoparticles and the mean side lengths of the cubic nanometer CuZnCo were measured using ImageJ 1.48r software. The chemical composition of the prepared nanoparticles was analyzed with energy-dispersive spectroscopy coupled with the employed SEM instrument. As expected, the greater the time of deposition, the more the particles grow, with a diameter ranging from 56 nm (for 30 s) to 105 nm (for 200 s). But what was unanticipated is that the content of the three metals in the formed nanoparticles is greatly influenced by the deposition time. In all the CuZnCo deposits obtained in acidic media, the Cu content was predominant and varied between 70% and 95%, increasing with the time of deposition and nanoparticle size, respectively. This behavior can be ascribed to the competitive relationship between Cu, Zn, and Co atoms during the deposition process since the Cu content in the deposits exceeds that of Zn and Co, indicating a preference for the induced deposition of Cu as compared to Zn and Co. 

**Figure 8:**
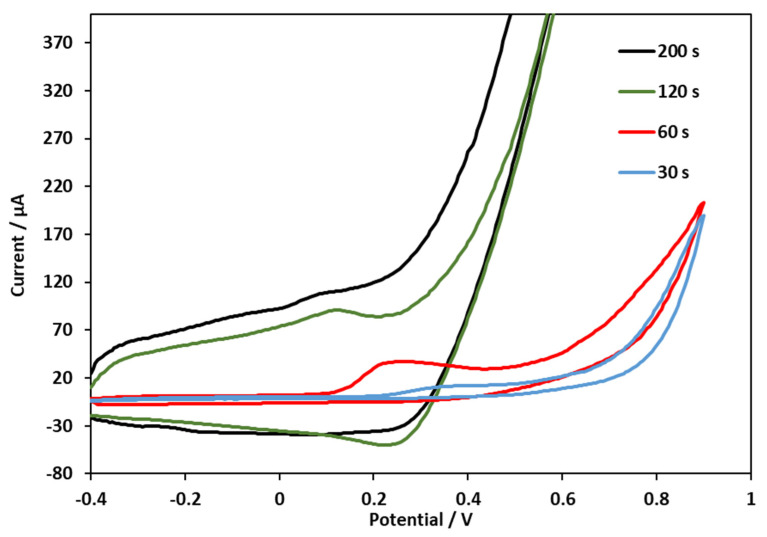
Electrochemical oxidation of 0.25 mM Trp in 0.05 M KOH on trimetallic CuZnCo deposited for different times: 30 s, 60 s, 120 s, and 200 s. Scan rate: 0.1 V/s.

The influence of deposition time on the redox activity of the resulting nanoparticles was evaluated by measuring the voltametric response to 0.25 mM Trp in 0.05 M KOH for each type of CuZnCo deposited for different times (Figure 8). The anodic peak current increases considerably from nanoparticles deposited 30 s to nanoparticles obtained for 60 s. With a further increase in deposition time and particle size, the capacitive current of the CuZnCo-based electrode increases, and consequently the oxidation peak of Trp becomes hardly quantifiable. Therefore, at nanoparticles deposited for 200 s, it is not even clearly defined at the tested concentration. This can be explained by the excessive growth of nanoparticles, changing their shape from spherical to cubic and thickening the entire composite layer. The results proved that in addition to the composition of the material, the nanoparticle size plays a major role in determining the optimal electrocatalytic effect to Trp oxidation.

### 2.3. Influence of Scan Rate on the Voltametric Response of Tryptophan

**Figure 9:**
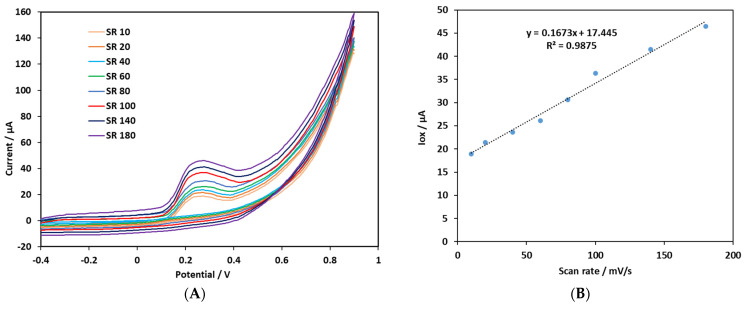
(**A**) Cyclic voltammetry curves of 0.25 mM Trp in 0.05 M KOH on trimetallic CuZnCo electrode at different scan rates: 10, 20, 40, 60, 80, 100, 140, and 180 mV/s. (**B**) Dependence of the oxidation peak current on the scan rate.

The relationship between peak current and scan rate was investigated to understand the electrochemical mechanism of the CuZnCo-modified electrode. Figure 9A shows the recorded CV curves for 0.25 mM Trp in 0.05 M KOH (pH 12) at different scan rates from 10 to 180 mV/s. According to Figure 9B, the oxidation peak current increases linearly with the scan rates, suggesting that the electrocatalytic oxidation reaction of Trp on the CuZnCo electrode is an adsorption-controlled process.

The linear regression equation is Ip (μA) = 17.445 + 0.1673v (mV s^−1^), with a correlation coefficient of R = 0.9875. In addition, the oxidation peak potential slightly shifted to more positive potentials with an increasing scan rate, confirming the kinetic limitation in the electrochemical reaction. 

### 2.4. Analytical Performances of the CuZnCo-Modified Electrode toward Tryptophan

**Figure 10:**
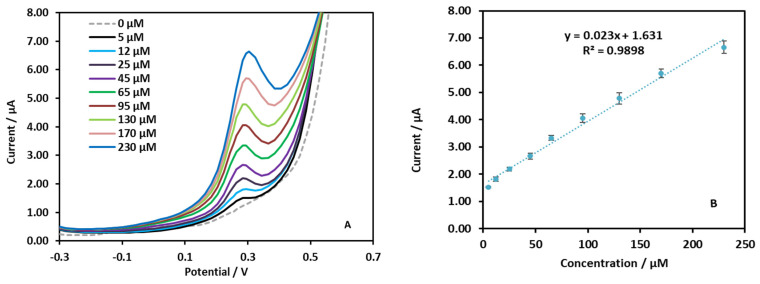
(**A**) DPV of Trp in the concentration range from 5 to 230 µM on CuZnCo-modified electrode in 0.05 M KOH solution. (**B**) The dependence of peak current on Trp concentration (investigated concentration range: 5–230 µM).

The analytical performance of the CuZnCo electrode was evaluated using DPV in 0.05 M KOH containing Trp at different concentrations under the same conditions. As depicted in Figure 10A, with the augmentation of the concentration of Trp, the peak currents increased, while Figure 10B shows the calibration plot of the current peak increasing linearly with the concentration of Trp with the corresponding equation.

Further, the limit of detection (*LOD*) was calculated according to *LOD* = 3 s/slope, where *s* is the standard deviation of peak currents. The calculated value of *LOD* was 1.1 µM. The obtained sensitivity is high enough to respond to the clinically relevant urinary Trp level in a healthy adult, which is in the range of 20–70 μM, and to the blood level of Trp, which is in the range of 45.5–83 μM. This level can even increase to 0.1–10 mM in people suffering from some inborn metabolic diseases [61]. The analytical performance of the proposed trimetallic CuZnCo nanoparticle electrodes compares favorably with that of other metallic-nanoparticle-based electrodes previously reported in literature (Table 2). Moreover, the detection of Trp occurs at a lower potential compared to that of all the other modified electrodes cited herein.

### 2.5. Electrode Stability and Repeatability

The operational stability of the CuZnCo electrode was assessed by using the same electrode for five repetitive measurements in the same solution containing 65 µM Trp in 0.05 M KOH, using DPV in the potential range of −0.3–0.7 V. The oxidation response decreased by 15% from the first measurement to the fifth, probably due to the adsorption of Trp and reaction products. The results indicate that the modified electrode is more adequate as a disposable sensor for the single-use determination of Trp.

In addition, the electrode-to-electrode reproducibility was evaluated using five electrodes prepared independently in the same conditions, and an RSD of 2.5% was obtained (calculated for the response to 65 μM Trp in 0.05 M KOH). These experimental results indicate that the proposed electrochemical sensor possesses good reproducibility. 

### 2.6. Effect of pH Values

**Figure 11:**
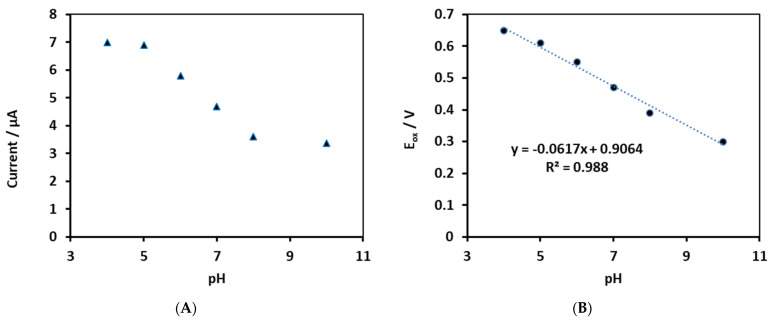
(**A**) The dependence of the oxidation peak current of 65 µM Trp on pH values of 0.1 Mf PBS. Scan rate: 100 mV/s. (**B**) The relation between peak potential of Trp and pH.

The effect of pH on the electrochemical responses of Trp on the CuZnCo-modified electrode was examined using DPV for 65 µM Trp in 0.1 M PBS. The plots of peak current against pH over the range of 4–10 is shown in Figure 11A. The maximum peak current was observed at pH 4; after this point, the oxidation peak current gradually decreased when the pH increased. Moreover, the oxidation potential of Trp on CuZnCo shifted negatively with the increase in pH, which means the oxidation process is associated with proton transfer. The linear dependence between potentials and pH for Trp is characterized by the following regression equation:

Ep (V) = −0.061pH + 0.906 (R = 0.988)

A slope of 0.061 V/pH is close to the theoretical value of 59 mV/pH for a two-electron/two-proton process. This suggests that the electrochemical reaction of Tryptophan on the CuZnCo-modified electrode is a two-electron transfer process coupled with two-proton transfer steps. 

### 2.7. Interference Studies

**Figure 12:**
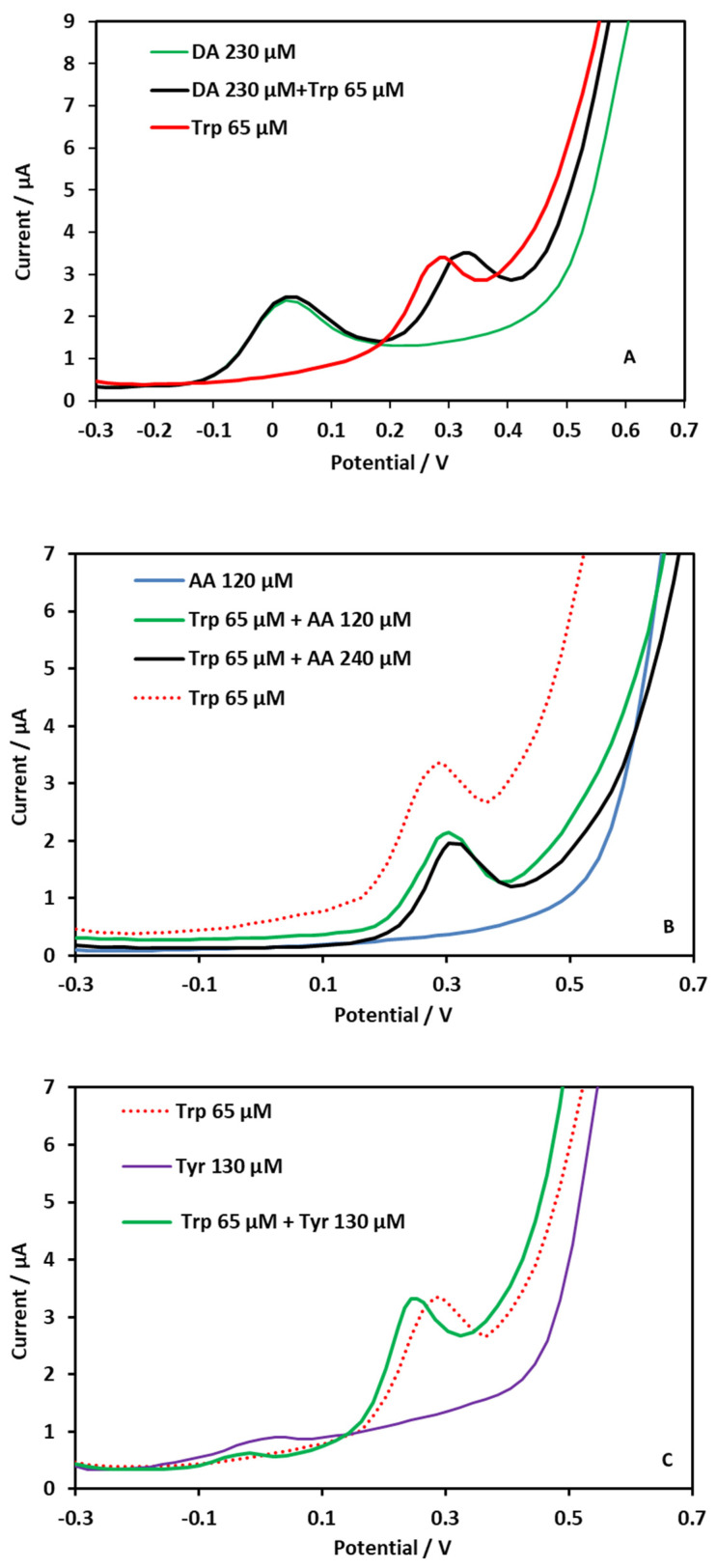
(**A**) Comparison of 65 µM Trp alone and in the presence of 230 µM DA; (**B**) comparison of 65 µM Trp alone and in the presence of 120 and 240 µM of AA; (**C**) tryptophan, tyrosine, and their mixture in 0.05 M KOH solution (pH 12).

Ascorbic acid (AA) and dopamine (DA) are two kinds of important biological substances often coexisting with Trp in human fluids and clinical analysis. Thus, an evaluation of the possible interferences of AA and DA was performed to test the selectivity of the CuZnCo-based electrode. Figure 12A shows the DPV of 230 µM DA coexisting with 65 µM Trp and compared to the response of the pure 65 µM Trp sample in 0.05 M KOH solution at pH 12. Dopamine was electrochemically oxidized on the CuZnCo electrode, and the corresponding peak appears at a lower potential than that of L-tryptophan (+0.05 V). When Trp is added to a high concentration of DA, the oxidation peaks of the two compounds appear distinctly separated, and their peak current is not affected. Only the oxidation peak for Trp is slightly shifted to a higher potential (with 30 mV), possibly due to the slight change in pH, since dopamine electrooxidation generates quinone and protons in the media, thus changing the pH in the close vicinity of the electrode surface and of Trp.

In the case of AA (Figure 12B), a shift in the background current can be observed. AA does not present an oxidation peak in the examined potential range and under the given conditions, probably due to the high pH of the solution. The oxidation of Trp in the presence of low and high concentrations of AA was not affected in terms of the height of the peak, but a shift in the background current can be observed in the presence of AA.

Along with tryptophan, Tyrosine (Tyr) is also an essential amino acid in human bodies, acting as the precursor for the synthesis of catecholamines such as dopamine and serotonin. Since Tyr is well known to have electrochemical activity, its possible interference in the detection of Trp was thus evaluated. Figure 12C shows the DPV for tyrosine and tryptophan alone and mixed together (with a twofold higher concentration of Tyr). A small shoulder can be seen at around 0 V for Tyr, and this is even smaller when Tyr is mixed with Trp, whereas the oxidation peak of Trp is not altered by the presence of Tyr. The results indicate the excellent selectivity of the sensor for Trp since no significant interference was observed, and this can be explained by the different mechanisms of the electrochemical oxidation of the tested compounds: the two-electron oxidation process of tryptophan occurred at the double bond in the pyrrole ring rather than the benzene ring.

### 2.8. Real Sample Analysis

**Figure 13:**
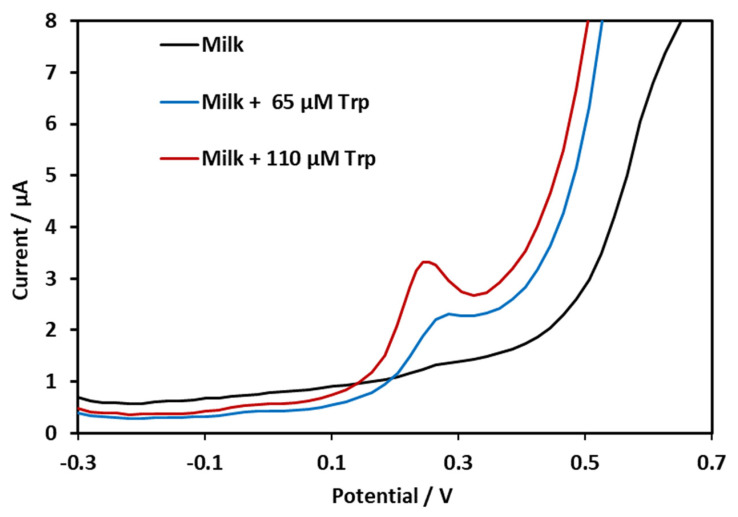
DPV recorded on CuZnCo-modified electrode for two standard additions of Trp (65 and 110 µM) in milk.

To determine whether the CuZnCo electrode was suitable for tryptophan detection in real samples, differential pulse voltammetry measurements were taken in milk samples using standard addition methods. The milk was bought from the local market (3.5% fat) and prepared via 10-fold dilution in 0.1 M PBS (pH 7). Two different concentrations of tryptophan were added to the diluted milk sample, and DPV measurements were conducted in 0.05 M KOH (Figure 13); the results are summarized in Table 3. The CuZnCo-electrode showed a good recovery of 96.1% for a low concentration of Trp and 94.5% for a high concentration of Trp. The obtained results show that the trimetallic-nanoparticle-based-electrode was suitable for the analysis of L-tryptophan in real milk samples.

## 3. Materials and Methods

### 3.1. Reagents

Pure L-tryptophan, L-tyrosine, and L-ascorbic acid were purchased from Sigma-Aldrich (St. Louis, MO, USA). 3-hydroxytyramine hydrochloride (dopamine) (98.0%) was supplied by TCI. CoCl_2_ anhydrous (97%), CuSO_4_·5H_2_O, ZnSO_4,_ KCl, KOH, and H_2_SO_4_ (96.0%) were purchased from Chemical Company (Iasi, Romania).

### 3.2. Apparatuses and Methods

Electrochemical experiments were carried out using a Multi Potentiostat/Galvanostat DRP-STAT8000-μStat8000 produced by DropSens, Asturias, Spain, and three-electrode systems with a planar configuration of screen-printed electrodes (SPEs) fabricated by and purchased from Metrohm DropSens (Oviedo, Spain). Working (4 mm diameter) and counter electrodes were made of carbon. Amperometry was used for electrodeposition of mono-, bi-, and trimetallic nanoparticles by applying a constant potential of −1.2 V for 60 s. To study the influence of deposition time on the morphology and redox behavior of nanoparticles, CuZnCo nanoparticles were deposited using four different deposition times: 30, 60, 120, and 200 s. 

Two voltametric methods, cyclic voltammetry (CV) and differential pulse voltammetry (DPV), were used in the investigation. DPV was conducted under the following conditions: pulse amplitude of 50 mV, pulse time of 50 ms, and a step potential of 5 mV within the potential range of −0.3–0.7 V (*vs*. Ag/AgCl); CV measurements were performed in a potential range specified for each experiment, with a scan rate of 0.1 V/s. The study of scan rate was performed in the range from 10 to 180 mV/s.

Electrochemical impedance spectroscopy tests (EIS, 200 kHz–1 Hz, 5 mV/s of ac voltage) were performed using an electrochemical workstation (MultiPalmSens4—Multichannel Potentiostat/Galvanostat/Impedance analyzer, PalmSens BV, Houten, The Netherlands) at room temperature.

The micrographs of the coatings were captured with a Quanta 200-FEI scanning electron microscope, and the ratio of metallic components of the nanoparticles was measured via energy-dispersive spectrum (EDX) coupled with SEM by using area mapping. Size distributions of DCFs were established by measuring the diameter of 50 particles using ImageJ 1.48r, a free image-processing program developed by the National Institute of Health (NIH).

X-ray photoelectron spectroscopy (XPS) analysis of the sample was carried out using a SPECS spectrometer equipped with monochromatized Al Kα (1486.61 eV). The anode radiation source was operated at 250W (12.5 kV × 20 mA). The high-resolution spectra were recorded at pressures lower than 2 × 10^−9^ mbar with 30 eV pass energy. Using a neutralizer flood gun operated at 1 eV × 0.1 mA, partial charge compensation was achieved. The parameters had to be optimized in order to obtain the C 1s peak of the sample at 284.60 ± 0.01 eV. 

### 3.3. Modification of the Electrode

The metallic nanoparticles were deposited onto the working electrode surface using the chronoamperometry technique, which consists of applying a fixed potential (−1.2 V) during a certain time specified for each experiment, in a solution containing 10 mM of the corresponding metallic salt prepared in 0.5 M of H_2_SO_4_. The mono-, bi-, and trimetallic deposits were electrochemically prepared from bath solutions containing copper sulfate pentahydrate (CuSO_4_), zinc sulfate (ZnSO_4_), and cobalt chloride (CoCl_2_) as the sources of Cu, Zn, and Co species, using the ratio shown in Table 4. The generated electrodes were rinsed thoroughly with distilled water.

## 4. Conclusions

A tryptophan electrochemical sensor was successfully constructed by employing trimetallic CuZnCo nanoparticles as an advanced electrocatalyst, which was prepared via electrodeposition on a carbon electrode substrate. This method is easy to use and can be adapted for the production of nanoparticle systems of other metals. The synergistic effects of the components endow the sensor with exceptional properties towards tryptophan oxidation with a wide linear range (5–230 μM), a low detection limit (1.1 µM), high sensitivity, and preferable selectivity. Also, a remarkable negative shift in the oxidation peak potentials to lower values was obtained. The sensor is selective, inexpensive, reproducible, and disposable, as well as simple to manufacture and operate.

## Figures and Tables

**Table 1:** The parameters of the oxidation peak of Trp on mono-, bi-, and trimetallic-nanoparticle-modified electrodes.

Electrode	Eox (V)	Iox (µA)
Bare	0.5	11.1
ZnNp	0.45	17.3
CoNp	0.4	18.6
CuNp	0.38	21.9
CuZnNp	0.35	25.9
CuCoNp	0.34	25.6
CuZnCoNp	0.26	37.18

**Table 2:** Comparison of analytical parameters of different metallic-nanoparticle-modified electrodes for tryptophan detection.

Electrode	E_ox_ of Trp	Detection Technique	Sensitivity (µA/µM)	Linear Ranges (µM)	*LOD* (µM)	Ref
Flower-like CeVO_4_/GCE	0.82 V	DPV	0.0289	0.1–94	0.024	[16]
CuCoHCF/graphite electrode	0.65 V	Amperometry	0.046	10–900	6.0	[18]
Nafion/TiO_2_-Graphene/GCE	0.79 V	DPV	0.0759	5–140	0.7	[19]
CeO_2_-RGO/GCE	0.6 V	DPV	0.5635	0.2–25	0.08	[17]
Cu_2_O–ERGO/GCE	0.92 V	SWV	3.159	0.02–20	0.01	[22]
Pd–Cu@Cu_2_O/N-RGO/GCE	0.78 V	DPV	0.3923	0.01–40.0	1.9	[23]
Co_3_O_4_/Graphene/Nafion/GCE	0.77 V	SWV	0.1859	0.05–10	0.01	[46]
NiCoO_2_/C modified GCE	0.54 V	LSV	0.0059	10–1000	1.8	[62]
CuZnCo/carbon electrode	0.26 V	DPV	0.023	5–230	1.1	This work

**Table 3:** Determination of L-tryptophan in milk in 0.05 M KOH solution using CuZnCo-modified electrode (N = 3).

Milk Sample	Added Trp (µM)	Found Trp (µM)	Recovery (%)	RSD
1	65	62.5 ± 0.68	96.1	5.9
2	110	104 ± 0.9	94.5	8.6

**Table 4:** Electrolyte compositions considered in the present study for metallic electrodeposition.

Solution/Electrode	CuSO_4_ 100 mM	ZnSO_4_ 100 mM	CoCl_2_ 100 mM	H_2_SO_4_ 0.5 M
Cu	0.5 mL	-	-	4.5 mL
Zn	-	0.5 mL	-	4.5 mL
Co	-	-	0.5 mL	4.5 mL
CuZn	0.5 mL	0.5 mL	-	4 mL
CuCo	0.5 mL	-	0.5 mL	4 mL
CuZnCo	0.5 mL	0.5 mL	0.5 mL	3.5 mL

## Data Availability

The data presented in this study are available in article.
